# Dataset on phytochemical screening, FTIR and GC–MS characterisation of *Azadirachta indica* and *Cymbopogon citratus* as reducing and stabilising agents for nanoparticles synthesis

**DOI:** 10.1016/j.dib.2018.08.133

**Published:** 2018-08-31

**Authors:** Oladotun P. Bolade, Anuoluwa A. Akinsiku, Alaba O. Adeyemi, Akan B. Williams, Nsikak U. Benson

**Affiliations:** a Department of Chemistry, Covenant University, Km 10 Idiroko Road, Ota, Nigeria; b Department of Biochemistry, Covenant University, Km 10 Idiroko Road, Ota, Nigeria

## Abstract

The dataset for this article contains phytochemical and FTIR data for three different extracts from two indigenous medicinal plants obtained from Ogun State, Southwest Nigeria and the GC–MS characterisation data for their ethanolic extracts. To obtain this data, the leaves of *Azadirachta indica* and *Cymbopogon citratus* were collected from the premises of Covenant University, Nigeria. The plants were dried, pulverized and extracted with ethanol, distilled water and ethanol:water (50:50), before phytochemical screening (qualitative and quantitative), FTIR and GC–MS analyses were carried out. The dataset provides insight into the presence of bioactive phyto-constituents such as polyphenols and tannins as potential precursors for green-based nanoparticle synthesis.

**Specifications Table**

**Table:** 

Subject area	*Chemistry, Biology*
More specific subject area	*Analytical Chemistry, Phytochemistry and Nanotechnology*
Type of data	*Table, figure, image*
How data was acquired	*Fourier Transform Infrared Spectroscopy (FTIR, AGILENT CARY 630)*
	*Gas Chromatography-Mass Spectroscopy (GC–MS, AGILENT 7890A GC/5977 MS)*
Data format	*Raw, analysed*
Experimental factors	*Phytochemicals (Fresh leaves were air-dried, pulverized, extracted with ethanol, distilled water, ethanol/water (1:1) and concentrated using rotary extractor under reduced pressure. Crude extracts were used for qualitative phytochemical analysis)*
	*FTIR (Range – 4000-650 cm*^*−1*^*, Resolution – 8 cm*^*−1*^*, Microlab PC software with ATR sampling unit)*
	*GCMS (Column - 30 mm × 0.25 mm ID × 0.25 μm film, Carrier gas - Helium, flow - 1.0 ml/min, electron ionization - 70 Ev, Software - Masshunter)*
Experimental features	*Phytochemical analysis of carbohydrates, tannins, saponins, flavonoids, alkaloids, anthocyanins, betacyanins, quinones, glycosides, cardiac glycosides, terpenoids, triterpenoids, phenols, coumarins, steroids, acids, FTIR scan of functional groups and GCMS scan of bioactive constituents.*
Data source location	*Ota, Nigeria*
Data accessibility	*Data included in this article*
Related research article	[1] P. Dubey, P. Sharma, V. Kumar, FTIR and GC–MS spectral datasets of wax from *Pinus roxburghii Sarg*. needles biomass, Data Brief. 15 (2017) 615–622. doi:10.1016/j.dib.2017.09.074.
	[2] K.M. Hammi, M. Hammami, C. Rihouey, D. Le Cerf, R. Ksouri, H. Majdoub, GC-EI-MS identification data of neutral sugars of polysaccharides extracted from *Zizyphus lotus* fruit, Data Brief. 18 (2018) 680–683. doi:10.1016/j.dib.2018.01.085.

**Value of the data**

• The dataset provides insight into the exact phyto-constituents, which are responsible for stabilization and reduction of metal ions during nanoparticles formation, thereby aiding proposition of mechanistic pathways for these reactions.
• The data provides information on the most potent of the locally selected plants for biosynthesis of nanoparticles using readily available indigenous plants in Southwest Nigeria.
• The methods used can be extended to other indigenous plants, forming a large database capable of informing researchers on the active plant(s) for nanoparticle synthesis.
• The dataset can be used for educational purposes, drug synthesis and multidisciplinary research. Similar data articles can be found in [1], [2].

## 1 Data

**Fig. 1:**
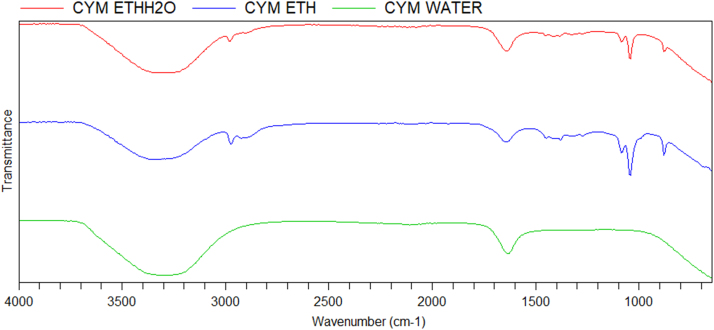
FTIR spectrum of three extracts of *Cymbopogon citratus* leaves.

**Fig. 2:**
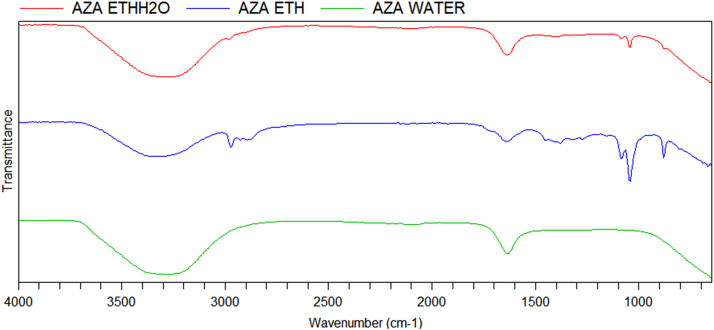
FTIR spectrum of three extracts of *Azadirachta indica* leaves.

**Fig. 3:**
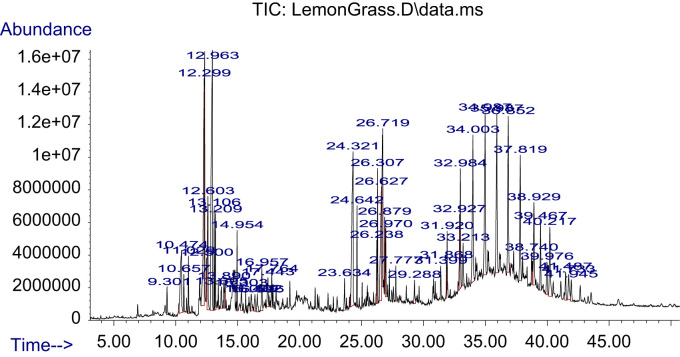
TIC of *Cymbopogon citratus* ethanolic extract.

**Fig. 4:**
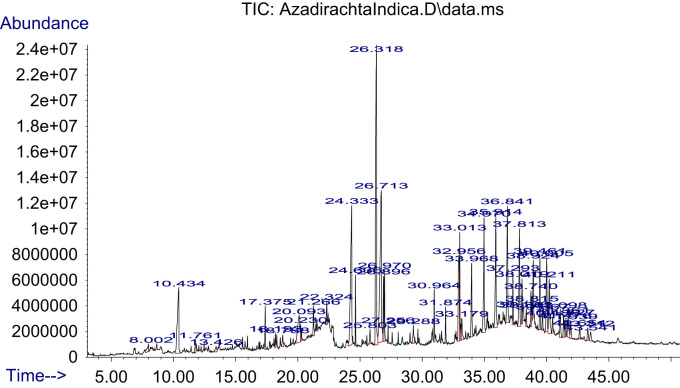
TIC of *Azadirachta indica* ethanolic extract.

The dataset on phytochemical screening of three extracts of *Azadirachta indica* and *Cymbopogon citratus* is presented in Table 1. FTIR spectra and data of different crude extracts of each plant are presented in Fig. 1, Fig. 2 and Table 2, respectively. GC–MS chromatogram/TIC of phyto-constituents of ethanolic extracts of plants and identification data of each constituent is provided in Fig. 3, Fig. 4 and Table 3, Table 4, respectively.

**Table 1:** Phytochemical screening of ethanol, water and ethanol/water (1:1) extracts of *Azadirachta indica* and *Cymbopogon citratus* leaves.

**Biochemicals / Inference**																
	**CHO**	**TAN**	**SAP**	**FLA**	**ALK**	**ANTHO**	**BETA**	**QUIN**	**GLY**	**CARD-GLY**	**TER**	**TRI-TERP**	**PHE**	**COU**	**STE**	**ACIDS**
***Ethanol extract***																
*C. citratus*		–	+	+	+	+	+	+	–	+	+	–	+	+	–	–
*A. indica*		–	+++	+	++	–	–	–	–	–	+	–	+	–	–	–

***Ethanol:water (1:1) extract***																
*C. citratus*		–	++	–	–	–	–	+	–	+	++	–	+	+	–	–
*A. indica*		–	+++	–	+	+	+	–	–	–	+	–	+	+	–	–

***Water extract***																
*C. citratus*		–	+	–	–	+	+	–	–	+	+	–	–	+	–	–
*A. indica*		–	+++	–	–	+	+	–	–	–	+	–	–	+	–	–

+ = trace amount; ++ = moderately present; +++ = highly present; - = absent.

CHO – Carbohydrates, TAN – Tannins, SAP – Saponins, FLA – Flavonoids, ALK – Alkaloids, ANTHO – Anthocyanins, BETA – Betacyanin, QUIN – Quinones, GLY – Glycosides, CARD-GLY – Cardiac.

Glycosides, TER – Terpenoids, TRI-TERP – Triterpenoids, PHE – Phenols, COU – Coumarins, STE – Steroids.

**Table 2:** FTIR frequency/intensity table for ethanol, water and ethanol/water extracts of *Cymbopogon citratus* and *Azadirachta indica* leaves.

**FTIR Absorption frequency (cm**^**-1**^**)/intensity**											
***C. citratus*****extracts**											
Ethanol	881 (m)	1048 (s)	1089 (m)	1275 (w)	1383 (w)	1640 (w)	2929 (w)	2974 (w)	3357(m,b)	–	–
Ethanol/water	881 (m)	1048 (m)	1089 (w)	–	–	1640 (m)	–	2891 (w)	3316 (s,b)	–	–
Water	–	–	–	–	–	1640 (m)	–	–	3316 (s,b)	–	–

***A. indica*****extracts**											
Ethanol	881 (m)	1048 (s)	1089 (m)	1383 (w)	1640 (w)	2892 (w)	2929 (w)	2974 (w)	3361(m,b)	–	–
Ethanol/water	881 (w)	1048 (w)	1089 (w)	–	1640 (m)	–	–	–	3264(s,b)	–	–
Water	–	–	–	–	1637 (m)	–	–	–	3331(s,b)	–	–

m – medium, s – strong, w – weak, b – broad.

**Table 3:** Identification of phyto-constituents in ethanolic extract of *C. citratus* leaves using GC–MS.

**Ret. time**	**Area %**	**IUPAC name of compound**	**Mol formular**	**Mol. wt.**
6.92	0.2126	Cyclohexane, 1,3,5-trimethyl-, (1.alpha.,3.alpha.,5.alpha.)-	C_9_H_18_	126.2392
9.14	0.2154	2-Acetylcyclopentanone	C_7_H_10_O_2_	126.1531
9.30	0.4783	1,6-Octadien-3-ol, 3,7-dimethyl-	C_10_H_18_O	154.2493
		OR Linalool		
10.47	2.6532	4H-Pyran-4-one, 2,3-dihydro-3,5-dihydroxy-6-methyl-	C_6_H_8_O_4_	144.1253
10.65	0.5139	Cyclooctane, ethenyl-	C_10_H_18_	138.2499
10.88	0.2167	Furan-2-carbohydrazide, N2-(1-methylhexylideno)-		
11.02	0.7758	7-Oxabicyclo[4.1.0]heptane, 1-methyl-4-(1-methylethenyl)-	C_10_H_16_O	152.2334
11.87	0.2083	Oxiranecarboxaldehyde, 3-methyl-3-(4-methyl-3-pentenyl)-	C_10_H_16_O_2_	168.2328
11.93	0.2702	Benzofuran, 2,3-dihydro-	C_8_H_8_O	120.1485
12.29	6.6959	2,6-Octadienal, 3,7-dimethyl-, (Z)-	C_10_H_16_O	152.2334
12.49	0.6401	Geraniol	C_10_H_18_O	154.2493
12.60	2.545			
12.96	9.4115	Citral	C_10_H_16_O	152.2334
13.10	1.1612	Epoxy-linalooloxide		
13.20	1.1323			
13.44	0.3872	Cyclopentane, (1-methylethyl)-	C_8_H_16_	112.2126
13.69	0.4762	2-Methoxy-4-vinylphenol	C_9_H_10_O_2_	150.1745
13.88	0.5025	Bicyclo[2.2.2]octan-1-amine		
13.98	0.3829	3-Cyclopropylcarbonyloxydodecane		
14.49	0.217	Triallylsilane	C_9_H_16_Si	152.3088
14.58	0.1855	3-Heptanol, 2-methyl-	C_8_H_18_O	130.2279
14.64	0.1943	1,5-Heptadiene, 3,3-dimethyl-, (E)-		
14.95	0.8738	Geranyl acetate	C_12_H_20_O_2_	196.2860
15.17	0.2231	Cyclopropanemethanol,.alpha.,2-dimethyl-2-(4-methyl-3-pentenyl)-, [1.alpha.(R*),2.alpha.]-		
15.30	0.514	Vanillin	C_8_H_8_O_3_	152.1473
15.65	0.3534	3,5-Heptadienal, 2-ethylidene-6-methyl-	C_10_H_14_O	150.2176
16.09	0.4413	Adamantane	C_10_H_16_	136.2340
16.40	0.4469	3-Cyclopentylpropionic acid, but-3-yn-2-yl ester		
16.59	0.4452	2-Propanol, 1,1,1-trichloro-2-methyl-	C_4_H_7_Cl_3_O	177.457
16.95	0.9541	2,6-Octadienal, 3,7-dimethyl-, (Z)-	C_10_H_16_O	152.2334
17.44	0.7992	3-Cyclohexene-1-acetaldehyde,.alpha.,4-dimethyl-	C_10_H_16_O	152.2334
17.76	0.6387	3-n-Propyl-2-pyrazolin-5-one	C_6_H_10_N_2_O	126.1564
17.85	0.3297	4-Methyl-5H-furan-2-one	C_5_H_6_O_2_	98.0999
18.11	0.3635	Dodecanoic acid	C_12_H_24_O_2_	200.3178
19.00	0.1681	1-Methyl-3-n-propyl-2-pyrazolin-5-one	C_7_H_12_N_2_O	140.1830
19.21	0.4101	Selina-6-en-4-ol		
19.76	0.1779	2-(2-Hydroxyethylthio)propionic acid		
20.89	0.2064	Phenylacetylformic acid, 4-hydroxy-3-methoxy-		
21.25	0.3042	Tetradecanoic acid	C_14_H_28_O_2_	228.3709
21.45	0.2678	Benzene, 1,1׳-ethylidenebis-	C_14_H_14_	182.2610
21.56	0.2124	Pyridine, 4-[(1,1-dimethylethyl)thio]-		
22.30	0.3018	p-Hydroxycinnamic acid, ethyl ester		
23.02	0.3948	2-Propenoic acid, 3-(4-hydroxy-3-methoxyphenyl)-	C_10_H_10_O_4_	194.1840
23.63	0.5611	p-Fluoroethylbenzene	C_8_H_9_F	124.1555
24.32	5.9637	n-Hexadecanoic acid	C_16_H_32_O_2_	256.4241
24.64	1.1441	Hexadecanoic acid, ethyl ester	C_18_H_36_O_2_	284.4772
25.05	0.3111	Heptadecanoic acid	C_17_H_34_O_2_	270.4507
25.47	0.3151	3-Methyl-2-butenoic acid, 2-tridecyl ester		
26.23	0.7845	Phytol	C_20_H_40_O	296.5310
26.30	1.6582	Diboroxane, triethyl[(4-methyl-2-pyridyl)amino]-		
26.62	3.8736	9,12-Octadecadienoic acid (Z,Z)-	C_18_H_32_O_2_	280.4455
26.71	3.6845	9,12,15-Octadecatrienoic acid, (Z,Z,Z)-	C_18_H_30_O_2_	278.4296
26.79	0.1683	Cyclooctene, 3-ethenyl-		
26.87	0.9543	Linoleic acid ethyl ester	C_20_H_36_O_2_	308.4986
26.97	0.988	Ethyl 9,12,15-octadecatrienoate		
27.11	0.2389	p-Menth-2-en-9-ol, trans-		
27.25	0.3594	Octadecanoic acid, ethyl ester	C_20_H_40_O_2_	312.5304
27.44	0.2377	5,9-Undecadien-2-one, 6,10-dimethyl-, (E)-	C_13_H_22_O	194.3132
27.57	0.2752	Naphtho[2,1-b:3,4-b׳]difuran, 2,3,8,9-tetrahydro-2,9-dimethyl-		
27.77	0.4932	Cyclohexanol, 5-methyl-2-(1-methylethenyl)-	C_10_H_18_O	154.2493
28.62	0.2172	1,6,10,14-Hexadecatetraen-3-ol, 3,7,11,15-tetramethyl-, (E,E)-	C_20_H_34_O	290.4834
29.28	0.4722	Eicosanoic acid	C_20_H_40_O_2_	312.5304
29.65	0.2219	Methyl 19-methyl-eicosanoate		
30.77	0.3075	9-Tricosene, (Z)-	C_23_H_46_	322.6113
31.86	0.527			
34.22	0.2424			
30.82	0.2058	Heptadecane	C_17_H_36_	240.4677
30.95	0.237	Hexadecanoic acid, 2-hydroxy-1-(hydroxymethyl)ethyl ester	C_19_H_38_O_4_	330.5026
31.39	0.5588	Dichloroacetic acid, heptadecyl ester		
31.91	0.6148	Hexacosane	C_26_H_54_	366.7070
32.79	0.2052	Cyclohexane, 1,1׳-[4-(3-cyclohexylpropyl)-1,7-heptanediyl]bis-	C_28_H_52_	388.7125
32.92	1.2801	1-Nonadecene	C_19_H_38_	266.5050
32.98	1.9114	Tetracosane	C_24_H_50_	338.6538
34.00	3.8594			
36.85	3.8693			
37.81	2.8508			
38.92	2.0079			
33.21	0.8589	Butane, 2,2-bis(5-acetyl-2-thienyl)-		
34.32	0.1678	Squalene	C_30_H_50_	410.7180
34.98	4.4543	Nonacosane	C_29_H_60_	408.7867
35.21	0.2288	Nonadecyl heptafluorobutyrate		
35.26	0.2462	Heptacosyl acetate		
35.93	4.1869	Triacontane	C_30_H_62_	422.8133
36.15	0.2301	Triacontyl acetate	C_32_H_64_O_2_	480.8494
37.29	0.4097	dl-.alpha.-Tocopherol	C_29_H_50_O_2_	430.7061
38.01	0.2942	Benzene, 1-nitro-4-(phenylthio)-	C_12_H_9_NO_2_S	231.270
38.37	0.3256	Campesterol	C_28_H_48_O	400.6801
38.74	0.5393	Stigmasterol	C_29_H_48_O	412.6908
38.81	0.3692	1,2,3,4-4H-Isoquinolin-1,3-dione, 4,4,5,6,8-pentamethyl-		
39.46	1.3421	.gamma.-Sitosterol	C_29_H_50_O	414.7067
39.97	0.8629	2-Furancarboxamide, N-[3-methyl-1-(phenylmethyl)-1H-pyrazol-5-yl]-		
40.21	1.9876	Tetratriacontane	C_34_H_70_	478.9196
40.48	0.2395	9,19-Cyclolanost-24-en-3-ol, (3.beta.)-	C_30_H_50_O	426.7174
41.09	0.4235	4-[5-(3,4-Diethoxy-benzyl)-[1,2,4]oxadiazol-3-yl]-furazan-3-ylamine		
41.48	0.6734	Cannabidiol	C_21_H_30_O_2_	314.4617
41.73	0.8292	Eicosane	C_20_H_42_	282.5475
43.54	0.3269			
41.94	0.5473	Cyclopropane-1-carboxamide, 2-butyl-N-(5,6,7,8-tetrahydro-7,7-dimethyl-5-oxoquinazolin-2-yl)-		
42.67	0.382	3-Methoxy-17beta-(O-nitrobenzoyloxy)-estra-1,3,5(10)-triene		
43.30	0.1722	2-(Acetoxymethyl)-3-(methoxycarbonyl)biphenylene

**Table 4:** Identification of phyto-constituents in ethanolic extract of *A. indica* leaves using GC–MS.

**Ret. time**	**Area %**	**IUPAC name of compound**	**Mol formular**	**Mol weight**
6.85	0.263	Thiazole, 4,5-dihydro-2-methyl-	C_4_H_7_NS	101.170
8.00	0.4441	2-Hexenoic acid	C_6_H_10_O_2_	114.1424
8.67	0.1883	2-Fluoro-5-methoxypyrimidine		
10.43	4.0847	4H-Pyran-4-one, 2,3-dihydro-3,5-dihydroxy-6-methyl-	C_6_H_8_O_4_	144.1253
10.88	0.1569	Isopropyl isothiocyanate	C_4_H_7_NS	101.170
11.42	0.2128	N-Aminopyrrolidine	C_4_H_10_N_2_	86.1356
11.76	0.4228	Benzofuran, 2,3-dihydro-	C_8_H_8_O	120.1485
11.83	0.2203	D-Alanine, N-allyloxycarbonyl-, decyl ester		
12.04	0.1826	2(1H)Pyrimidinone,4-amino-1,N-dimethyl-	C_6_H_9_N_3_O	139.1552
12.23	0.1549	2,6-Octadienal, 3,7-dimethyl-, (Z)-	C_10_H_16_O	152.2334
12.47	0.2256	Geraniol	C_10_H_18_O	154.2493
		OR 2,6-Octadien-1-ol, 3,7-dimethyl-, (E)-		
12.61	0.2274	N-[5-(3,4-Dimethoxy-benzyl)-[1,3,4]thiadiazol-2-yl]-3-fluoro-benzamide		
13.42	0.3247	Malic Acid	C_4_H_6_O_5_	134.0874
13.67	0.2593	2-Methoxy-4-vinylphenol	C_9_H_10_O_2_	150.1745
15.56	0.2434	1H-Cycloprop[e]azulene, 1a,2,3,4,4a,5,6,7b-octahydro-1,1,4,7-tetramethyl-, [1aR-(1a.alpha.,4.alpha.,4a.beta.,7b.alpha.)]-	C_15_H_24_	204.3511
15.75	0.1914	trans-Cinnamic acid	C_9_H_8_O_2_	148.1586
15.94	0.2789	.gamma.-Elemene OR γ-Elemene	C_15_H_24_	204.3511
17.37	0.8838	2-Hydroxy-1-(1׳-pyrrolidiyl)-1-buten-3-one		
17.96	0.1632	L-Proline, 1-acetyl-	C_7_H_10_NO_3_	156.1592
18.08	0.1893	Dodecanoic acid	C_12_H_24_O_2_	200.3178
18.19	0.3327	Cyclohexane, 1-ethenyl-1-methyl-2-(1-methylethenyl)-4-(1-methylethylidene)-	C_15_H_24_	204.3511
18.30	0.2576	Fumaric acid, cyclobutyl ethyl ester		
18.59	0.2391	Phosphine, methyl(1-methylethyl)phenyl-		
18.78	0.4281	Carbamic acid, methylphenyl-, ethyl ester	C_10_H_13_NO_2_	179.2157
20.09	2.4879	Ethyl.alpha.-d-glucopyranoside		
20.22	0.3267	.beta.-D-Glucopyranoside, methyl	C_7_H_14_O_6_	194.1825
20.29	0.2465	d-Glycero-l-gluco-heptose		
21.26	0.7649	2(1H)-Pyrimidinone, 5-methyl-		
21.54	0.2506	Sorbitol	C_6_H_14_O_6_	182.1718
22.32	0.5687	Piperidine, 1-(1-pentenyl)-		
22.53	0.2716	Galactitol	C_6_H_14_O_6_	182.1718
22.90	0.2182	Cyclohexane, 1,5-diisopropyl-2,3-dimethyl-		
23.91	0.2894	Palmitoleic acid	C_16_H_30_O_2_	254.4082
24.33	7.424	n-Hexadecanoic acid	C_16_H_32_O_2_	256.4241
24.40	0.1754	11-Oxa-tricyclo[4.4.1.0(1,6)]undecan-2-ol		
24.63	1.0398	Hexadecanoic acid, ethyl ester	C_18_H_36_O_2_	284.4772
25.54	0.1899	Heptadecanoic acid	C_17_H_34_O_2_	270.4507
25.80	0.4054	3-Heptanol, 3,5-dimethyl-	C_9_H_20_O	144.2545
26.31	11.5639	Phytol	C_20_H_40_O	296.5310
26.71	9.7212	9,12,15-Octadecatrienoic acid, (Z,Z,Z)-	C_18_H_30_O_2_	278.4296
26.89	1.5401	Octadecanoic acid	C_18_H_36_O_2_	284.4772
26.97	1.4276	Ethyl 9,12,15-octadecatrienoate		
27.25	0.329	Octadecanoic acid, ethyl ester	C_20_H_40_O_2_	312.5304
27.58	0.2923	Naphtho[2,1-b:7,8-b׳]difuran, 1,2,9,10-tetrahydro-2,9-dimethyl-		
28.06	0.2169	1-Heneicosyl formate	C_22_H_44_O_2_	340.5836
28.40	0.2843	Benzyl.beta.-d-glucoside		
29.02	0.213	Z,Z-8,10-Hexadecadien-1-ol acetate		
29.28	0.6416	Eicosanoic acid	C_20_H_40_O_2_	312.5304
29.65	0.2674	Methyl 19-methyl-eicosanoate		
29.75	0.1501	(1S,15S)-Bicyclo[13.1.0]hexadecan-2-one		
30.78	0.2073	Cyclotetradecane, 1,7,11-trimethyl-4-(1-methylethyl)-	C_20_H_40_	280.5316
30.82	0.245	Eicosane	C_20_H_42_	282.5475
30.96	1.0086	Hexadecanoic acid, 2-hydroxy-1-(hydroxymethyl)ethyl ester	C_19_H_38_O_4_	330.5026
31.07	0.254	Glycerol 1-palmitate	C_19_H_38_O_4_	330.5026
31.41	0.2199	Bis(2-ethylhexyl) phthalate	C_24_H_38_O_4_	390.5561
31.55	0.2299	Docosanoic acid	C_22_H_44_O_2_	340.5836
31.87	0.6983	Nonadecanoic acid, ethyl ester	C_21_H_42_O_2_	326.5570
32.64	0.2932	Cyclopentadecanone, 2-hydroxy-	C_15_H_28_O_2_	240.3816
32.70	0.2054	9,12,15-Octadecatrienoic acid, ethyl ester, (Z,Z,Z)-	C_20_H_34_O_2_	306.4828
32.95	3.8689	Ethanol, 2-(octadecyloxy)-	C_20_H_42_O_2_	314.5463
33.01	1.9763	Linolenic acid, 2-hydroxy-1-(hydroxymethyl)ethyl ester (Z,Z,Z)-	C_21_H_36_O_4_	352.5081
33.09	0.309			
33.17	0.8062	Benzene, 1,2-dimethoxy-4-nitro-	C_8_H_9_NO_4_	183.1614
33.48	0.1631	Fumaric acid, pent-4-en-2-yl tridecyl ester		
33.96	2.3197	Octacosane	C_28_H_58_	394.7601
34.31	0.1787	Squalene	C_30_H_50_	410.7180
34.96	3.6131	Nonacosane	C_29_H_60_	408.7867
35.19	0.1721	Octacosyl acetate	C_30_H_60_O_2_	452.7962
35.25	0.2752	1-Nonadecene	C_19_H_38_	266.5050
35.66	0.1803			
35.91	3.7204	Tetracosane	C_24_H_50_	338.6538
36.84	4.1428			
36.14	0.2142	Triacontyl acetate	C_32_H_64_O_2_	480.8494
36.21	0.165			
36.62	0.1643			
36.50	0.1834	.gamma.-Tocopherol	C_28_H_48_O_2_	416.6795
37.29	0.9935	Vitamin E	C_29_H_50_O_2_	430.7061
37.81	3.0663	Octadecane	C_18_H_38_	254.4943
38.92	1.5839			
38.01	1.0458	Pregn-4-ene-3,20-dione, 16-hydroxy-, (16.alpha.)-		
38.09	0.4445	2,6,10,14-Tetramethyl-7-(3-methylpent-4-enylidene) pentadecane		
38.36	0.4534	Campesterol	C_28_H_48_O	400.6801
38.74	0.8867	Stigmasterol	C_29_H_48_O	412.6908
38.81	0.3705	4-Cyclohexene-1,2-dicarboximide, N-butyl-, cis-		
39.46	2.0086	.gamma.-Sitosterol	C_29_H_50_O	414.7067
39.66	0.5223	4,4,6a,6b,8a,11,11,14b-Octamethyl-1,4,4a,5,6,6a,6b,7,8,8a,9,10,11,12,12a,14,14a,14b-octadecahydro-2H-picen-3-one		
40.00	2.5958	2-Furancarboxamide, N-(8-methyl-2H-[1,2,4]thiadiazolo[2,3-a]pyridin-2-ylidene)-		
40.21	2.2065	Eicosane	C_20_H_42_	282.5475
41.72	0.9181			
43.54	0.4271			
40.48	0.4548	4,22-Stigmastadiene-3-one	C_29_H_46_O	410.6749
41.09	0.9555	D:A-Friedoursan-3-one		
41.36	0.7263	Stigmast-4-en-3-one	C_29_H_48_O	412.6908
41.47	0.5	Cyclopropane-1-carboxamide, 2-butyl-N-(5,6,7,8-tetrahydro-7,7-dimethyl-5-oxoquinazolin-2-yl)-		
41.61	0.1877	Hexahydropyridine, 1-methyl-4-[4,5-dihydroxyphenyl]-		
41.93	0.6689	Cannabidiol	C_21_H_30_O_2_	314.4617
42.65	0.3853	1H-1,2,4-Triazole-5(4H)-thione, 4-allyl-3-(3-furyl)-		
43.00	0.1593	1,2-Bis(trimethylsilyl)benzene		
43.31	0.4858	Pyrido[2,3-d]pyrimidine, 4-phenyl-		
45.73	0.2579	2-(Acetoxymethyl)-3-(methoxycarbonyl)biphenylene		

## 2 Experimental design, materials and methods

### 2.1 Sample collection

Fresh leaves of two (2) indigenous plants namely *Cymbopogon citratus and Azadirachta indica* were collected in March 2018 from Covenant University, Nigeria. The leaf samples were thoroughly washed in distilled water before air-drying at room temperature for 21 days. Dried leaves were then pulverized and preserved in airtight containers until further use.

### 2.2 Sample preparation and characterisation

For phytochemical screening, 25 g of pulverized plant leaves was extracted with 125 mL of three solvents namely; ethanol, distilled water and ethanol/water (1:1) for 72 h. The plant extracts were filtered and concentrated using rotary evaporator under reduced pressure. Preliminary phytochemical analysis was carried out to test for the presence of tannins, saponins, flavonoids, alkaloids, anthocyanins, betacyanins, quinones, glycosides, cardiac glycosides, terpenoids, triterpenoids, phenols, coumarins, steroids and acids in all the three extracts following the standard test methods [3], [4].

Also, 10 g of each powdered plant material was extracted with ethanol, distilled water and ethanol/distilled water (1:1), respectively, for 72 h. The extracts were filtered and concentrated to 1 mL using BUCHI rotary evaporator under reduced pressure. Then, 1 mL of crude ethanolic, water and ethanol/water extracts were taken for FTIR analysis, while 1 mL ethanolic extracts were taken in amber GC vials for GC–MS analysis.

### 2.3 Fourier transform infrared spectroscopy analysis

The extracts were analysed using Agilent Cary 630 FTIR spectrometer equipped with Microlab PC software with ATR sampling unit with a resolution of 8 cm^−1^ and scan range of 4000 cm^−1^ to 650 cm^−1^.

### 2.4 Gas chromatography mass spectroscopy analysis

The GC–MS analysis was carried out using Agilent 7890 A gas chromatograph coupled with a 5977 A mass spectrometer. The temperature programme of the GC was maintained at an initial temperature of 50 °C with a hold for 1 min, followed by gradual increase to 300 °C at 7 °C/min for 14 min. 1 µL of each sample was injected in the split mode (split ratio 1:10). The identification of components was based on retention time on the capillary column and matching the GC mass spectra with the National Institute of Standards and Technology (NIST) library.
